# Editorial: Artificial intelligence in acute neurology

**DOI:** 10.3389/fneur.2025.1605735

**Published:** 2025-04-29

**Authors:** Tarun D. Singh, Alejandro A. Rabinstein

**Affiliations:** ^1^Department of Neurology and Neurosurgery, University of Michigan, Ann Arbor, MI, United States; ^2^Department of Neurology, Mayo Clinic, Rochester, MN, United States

**Keywords:** acute neurology, artificial intelligence, stroke, neurocritical care, neurohospitalist care

## Introduction

What if an algorithm could predict a life-threatening stroke hours before symptoms appear—or even before the patient realizes they are at risk? In acute Neurology, artificial intelligence (AI) is not just a tool; it can become the difference between life and death. No longer confined to theoretical algorithms or experimental models, AI is now influencing real-time decision-making, predictive analytics, and diagnostic accuracy. Its ability to analyze vast datasets, recognize complex patterns, and offer data-driven insights has transformed not just how we approach neurological emergencies, but also how we envision the future of patient care.

There is a lot of interest in AI's potential in automating image interpretation, identifying brain abnormalities with unprecedented accuracy, and accelerating diagnoses—thereby reducing the burden on clinicians and enhancing patient outcomes. This special edition, “*Artificial intelligence in acute neurology,”* reflects the rising global interest in this field and the growing momentum for integrating AI into clinical workflows. It features 17 high-quality manuscripts authored by 156 researchers from prestigious institutions across the United States, China, Germany, Japan, Singapore, India, the United Kingdom, Canada among other countries.

Since its launch, this Research Topic has garnered over 28,000 views and almost 10,000 downloads—demonstrating its global relevance and the increasing recognition of AI's transformative potential in clinical Neurology. Below, we highlight key insights, emphasize emerging trends, and discuss future directions for this dynamic and rapidly evolving field.

## Key highlights from the research contributions

AI's applications in acute Neurology span a wide range—from prehospital care, predictive modeling and diagnostic innovations to clinical decision support. The manuscripts in this special edition collectively give a glimpse onto how AI can redefine neurological care across these domains.

### Predictive models for neurological and systemic complications

AI-driven predictive models are enhancing clinicians' ability to anticipate complications such as early neurological deterioration, cerebral edema, and recurrent intracerebral hemorrhage. These tools enable earlier interventions, reducing morbidity, and improving long-term outcomes. Furthermore, AI's role extends beyond the brain—models predicting systemic issues like postoperative urinary retention and sepsis-associated encephalopathy illustrate AI's versatility in managing complex neurological patients holistically.

### Diagnostic innovations using imaging data

AI has shown remarkable potential in imaging analysis, from detecting subtle ischemic changes on CT scans to predicting stroke outcomes using radiomics and machine learning. Models integrating clinical and imaging data have improved diagnostic precision for conditions like post-concussive syndrome and intracranial hemorrhage, even in resource-limited settings. These advancements are democratizing access to expert-level diagnostics globally.

### AI in critical care and clinical decision-making

AI supports clinical decision-making by offering real-time, data-driven insights that enhance patient monitoring and risk assessment in Neurocritical care. Models predicting complications such as postoperative pneumonia in subarachnoid hemorrhage patients or identifying high-risk stroke patients underscore AI's potential to improve care pathways, reduce errors, and optimize resource allocation.

### Prehospital and global applications

AI's influence extends beyond hospital walls. In prehospital settings, AI models may assist in rapid stroke classification and triage, improving time-to-treatment metrics. Notably, models designed for resource-limited environments highlight AI's potential to bridge healthcare disparities, enabling frontline providers to make informed decisions even in the absence of specialist support.

Collectively, these studies demonstrate AI's capacity to enhance neurological care across the entire continuum—from the prehospital environment to critical care units—ushering in an era where data-driven insights complement clinical expertise.

### Ethical considerations in AI for acute neurology

While AI holds transformative potential, its integration into clinical practice raises critical ethical considerations. Addressing these challenges proactively is essential to ensure AI supports patient-centered, equitable, and safe neurological care.

### Transparency and accountability

Many AI models operate as “black boxes,” making it difficult to understand how specific predictions are generated. Ensuring transparency in AI algorithms is vital for building clinician trust. Explainable AI (XAI) techniques, such as SHapley Additive exPlanations (SHAP), help demystify these models by illustrating how various data inputs influence outcomes.

### Bias and fairness

AI models are only as unbiased as the data they are trained on. If datasets lack diversity, models may perpetuate healthcare disparities, particularly for underrepresented populations. Ensuring fairness requires deliberate efforts to diversify training datasets and develop algorithms that perform consistently across different demographic groups.

### Data privacy and security

AI relies on large datasets, often aggregated from multiple sources. Protecting patient privacy while enabling data-driven innovation is a delicate balance. Privacy-preserving techniques like federated learning offer promising solutions, allowing models to learn from decentralized data without compromising individual privacy.

### Clinical responsibility

AI is a decision-support tool—not a replacement for clinical judgment. Clear guidelines must define the roles and responsibilities of clinicians when using AI-driven recommendations. In cases of adverse outcomes, questions around liability—whether it rests with the developer, the clinician, or the institution—must be addressed within legal and ethical frameworks.

By fostering transparency, fairness, and accountability, we can harness AI's potential responsibly, ensuring it complements rather than complicates clinical care.

## Future directions for AI in acute neurology

As AI continues to evolve, several emerging trends are poised to shape the future of acute neurology.

### Personalized neurological care

AI's ability to analyze large, complex datasets opens the door to truly personalized medicine. Integrating genomic, imaging, and clinical data, AI can predict individual responses to treatments, tailoring interventions for conditions like stroke, traumatic brain injury, and neuroinflammatory diseases.

### Real-time decision support

The future lies in real-time AI applications that continuously monitor patients in neurocritical care units. These systems can detect early signs of clinical deterioration—such as impending cerebral edema, vasospasm in aSAH patients or intracranial hypertension in traumatic brain injury (TBI)—allowing for rapid interventions before irreversible damage occurs.

### Adaptive learning models

Unlike traditional algorithms that remain static after deployment, adaptive AI models can learn from new data, continuously refining their predictions. This dynamic capability ensures that AI systems evolve alongside advances in medical knowledge and changes in patient populations.

### Integration with brain-computer interfaces

The convergence of AI with BCIs offers exciting possibilities, particularly for patients with severe neurological impairments. AI-enhanced BCIs could improve communication and motor control for individuals with conditions like locked-in syndrome or advanced neurodegenerative diseases.

### Global health and resource-limited settings

AI's potential to improve care extends beyond high-resource settings. Models designed for use in resource-limited environments—where access to specialists is scarce—can democratize neurological care, providing diagnostic and decision-support tools to frontline healthcare workers worldwide.

While the promise of AI in acute neurology is vast, realizing its full potential will require ongoing collaboration across disciplines, continuous evaluation, and a commitment to ethical, patient-centered care.

## Call to action

The integration of artificial intelligence into acute neurology represents a paradigm shift—one that holds the promise of transforming patient outcomes through enhanced diagnostics, predictive analytics, and personalized care. However, realizing this potential requires coordinated efforts from clinicians, researchers, policymakers, and technologists.

### Foster interdisciplinary collaboration

AI's successful integration into clinical practice depends on partnerships between neurologists, data scientists, ethicists, and engineers. Collaborative research will ensure that AI models are not only technically robust but also clinically meaningful.

### Promote ethical AI development

Stakeholders must prioritize fairness, transparency, and accountability in AI development. Ethical frameworks should guide data collection, model training, and clinical deployment to ensure that AI benefits all patients equitably.

### Invest in education and capacity building

Healthcare professionals need ongoing education to effectively leverage AI tools. Training programs should focus on understanding AI's capabilities and limitations, fostering a culture of informed adoption rather than passive reliance.

By embracing these principles, the neurological community can lead the way in shaping an AI-driven future that prioritizes patient wellbeing, clinical excellence, and ethical integrity.

## Conclusion

The contributions in this special edition underscore the transformative potential of AI in acute neurology. From predictive modeling and diagnostic innovations to ethical considerations and future directions, the 17 manuscripts featured here reflect a vibrant, global effort to harness AI's power for the benefit of patients worldwide.

However, the journey toward full integration is not without challenges. Ethical dilemmas, data privacy concerns, and the need for interdisciplinary collaboration will shape how AI evolves within clinical practice. By addressing these challenges head-on, we can ensure that AI serves as a tool for empowerment—enhancing clinical decision-making, improving patient outcomes, and advancing the frontiers of neurological care.

We extend our deepest gratitude to the authors, reviewers, and editorial board members for their dedication and contributions. With over 16,000 views and 8,000 downloads, this Research Topic has already made a meaningful impact, sparking conversations, and inspiring innovation across the neurological community.

The future of acute Neurology will be defined not just by the data we collect and the AI models we develop, but primarily by how intelligently we use them. Now is the time to lead, innovate, and transform. And it is the time to decide how AI can work for us and not replacing us. We will always need empathetic and judicious neurologists. AI should support their clinical tasks and reduce cognitive biases, but for the sake of our patients those tasks should remain an eminently human endeavor.

